# Targeting Pro-Oxidant Iron with Exogenously Administered Apotransferrin Provides Benefits Associated with Changes in Crucial Cellular Iron Gate Protein TfR in a Model of Intracerebral Hemorrhagic Stroke in Mice

**DOI:** 10.3390/antiox12111945

**Published:** 2023-10-31

**Authors:** Alexia García-Serran, Jesús Ordoño, Núria DeGregorio-Rocasolano, Marc Melià-Sorolla, Karla Odendaal, Octavi Martí-Sistac, Teresa Gasull

**Affiliations:** 1 Cellular and Molecular Neurobiology Research Group, Fundació Institut d’Investigació en Ciències de la Salut Germans Trias i Pujol (IGTP), Universitat Autònoma de Barcelona (UAB), 08916 Badalona, Catalonia, Spain; agarcias@igtp.cat (A.G.-S.); jesus.ordono@imdea.org (J.O.); ndgregorio@igtp.cat (N.D.-R.); mmelia@igtp.cat (M.M.-S.); kk.o@btinternet.com (K.O.); 2 School of Biosciences, University of Cardiff, Cardiff CF10 3AT, UK; 3 Department of Cellular Biology, Physiology and Immunology, Universitat Autònoma de Barcelona, 08193 Bellaterra, Catalonia, Spain

**Keywords:** ischemic stroke, intracerebral hemorrhage, iron, oxidation, apotransferrin, mouse

## Abstract

We have previously demonstrated that the post-stroke administration of iron-free transferrin (apotransferrin, ATf) is beneficial in different models of ischemic stroke (IS) through the inhibition of the neuronal uptake of pro-oxidant iron. In the present study, we asked whether ATf is safe and also beneficial when given after the induction of intracerebral hemorrhage (ICH) in mice, and investigated the underlying mechanisms. We first compared the main iron actors in the brain of IS- or collagenase-induced ICH mice and then obtained insight into these iron-related proteins in ICH 72 h after the administration of ATf. The infarct size of the IS mice was double that of hemorrhage in ICH mice, but both groups showed similar body weight loss, edema, and increased ferritin and transferrin levels in the ipsilateral brain hemisphere. Although the administration of human ATf (hATf) to ICH mice did not alter the hemorrhage volume or levels of the classical ferroptosis GPX4/system xc- pathways, hATf induced better neurobehavioral performance, decreased 4-hydroxynonenal levels and those of the second-generation ferroptosis marker transferrin receptor (TfR), and restored the mRNA levels of the recently recognized cytosolic iron chaperone poly(RC) binding protein 2. In addition, hATf treatment lowered the ICH-induced increase in both endogenous mouse transferrin mRNA levels and the activation of caspase-2. In conclusion, hATf treatment provides neurobehavioral benefits post-ICH associated with the modulation of iron/oxidative players.

## 1. Introduction

A global consensus exists throughout the literature reporting the detrimental effects of systemic iron overload in ischemic stroke, and the protective effect of iron chelators and the iron-binding protein apotransferrin (ATf) in ischemic stroke models [1,2,3] and ischemic stroke patients [4]. Moreover, iron chelators and ATf have been studied as neuroprotectors in hemorrhagic stroke models. Chen-Roetling J et al. reported the protective effect of ATf in cortical neurons [5], and deferoxamine has been reported to be beneficial in hemorrhagic stroke patients [6,7]. In preclinical studies, our laboratory has shown that ATf, the iron-free form of transferrin (Tf), is a potent neuroprotective agent in ischemic stroke because it reduces the saturation of blood transferrin with iron (TSAT) and inhibits the entry of iron-Tf into the neurons [3].

Intracerebral hemorrhage (ICH), besides the damage it induces through an increase in intracranial pressure derived from a sudden accumulation of spilled blood in the brain parenchyma, brings about toxic effects produced by substances of the leaked blood [8,9]. In particular, the breakdown of erythrocytes leads to the release of hemoglobin, from which heme and, ultimately, high amounts of iron are released to the cerebral parenchyma [10,11,12]. These compounds lead to oxidative stress, blood–brain barrier disruption, and the activation of different death pathways in the hematomal and perihematomal regions that lead to neuronal death [13,14].

Ferroptosis, a non-apoptotic and iron-dependent programmed cell death [15], has been described as playing a crucial role, especially at early timepoints, in the ICH model induced by collagenase [16]. Nevertheless, contrasting results have been reported regarding the effect of ICH on the expression of some iron- and classical ferroptosis-related proteins [17,18,19]. Such discrepancies might by due, at least in part, to the severity of the model and experimental design/time course or the brain region analyzed. Alternatively, beyond the classical ferroptotic mechanisms directly linked to iron and loss of reactive oxygen species (ROS) detoxifying potential, the ferroptosis field is evolving and the most recently discovered ferroptotic pathways might have specific roles that have not yet been identified.

To date, the whole picture of the mechanisms driving hemorrhagic stroke damage is still far from complete. Specifically, the therapeutic potential and safety profile of ATf in hemorrhagic stroke is not known, although ferroptosis, an iron-mediated type of cell death, has been previously suggested to be implicated [16]. We hypothesized that ATf, which has demonstrated benefits in ischemic stroke, could reduce the detrimental consequences of ICH and, thus, is a potential pre-hospital and pre-triage frontline treatment for stroke patients. The present work aimed to investigate (1) the effect of early treatment with ATf on the hematoma size, antioxidant status, and neurological outcome in a mouse model of ICH, and (2) the effect of ICH and ATf on the levels and expression of iron- and ferroptosis-related molecules relevant in stroke.

## 2. Materials and Methods

### 2.1. Animals

Adult 9-week-old male C57BL/6J mice purchased from Charles River Laboratories were used in this study. They were housed in controlled standard conditions of temperature, humidity, and photoperiod, and had food and water ad libitum. The animal experiments were approved by the Animal Research Ethics Committee (CEEA) of the Germans Trias i Pujol Research Institute (IGTP) and the Catalan Government (references 11182 and 11131) and were conducted in a randomized manner and according to international guidelines (EU Directive 2010/63/EU) at the Comparative Medicine and Bioimage Centre of Catalonia (CMCiB). We followed the ARRIVE guidelines and were committed to the 3Rs of laboratory animal research. As postoperative care and until complete recovery, the operated animals were placed onto a heating pad and 0.8 mL subcutaneous sterile saline was injected. Euthanasia was performed by cervical dislocation after completion of the experiments.

### 2.2. Hypoxic/Ischemic Stroke Model (IS) 

The mice (n = 12) were exposed to transient unilateral brain ischemia followed by a global hypoxia procedure adapted from Guan et al. [20]. In brief, the mice were anesthetized with isoflurane (4% for induction and 1.5% for maintenance) in a 30–70% mixture of oxygen (O_2_) and nitrous oxide (N_2_O) and the right common carotid artery (CCA) was transiently occluded by ligature (using a 5-0, black braided silk non-absorbable suture) (Ethicon, LLC.; San Lorenzo, Puerto Rico). The mice were allowed to recover with free access to food and water for 2 h and then placed in a hypoxia chamber at 35.5 °C for 20 min, ventilated with a humidified gas mixture of 8% O_2_/92% N_2_ at an airflow rate of 1 L/min. Then, under general anesthesia with isoflurane, the suture occluding the CCA was removed, blood flow restoration in the CCA was verified de visu, and the wound was sutured with a 4-0, black braided silk non-absorbable suture (Silkam, B.Braun; Rubí, Spain).

### 2.3. Intracerebral Hemorrhage Stroke Model (ICH)

The ICH procedure was adapted from Klebe et al. [21]. Briefly, the mice were anesthetized with isoflurane (4% for induction and 1.5% for maintenance) in a 30/70 mixture of O_2_/N_2_O and placed in a stereotaxic frame (Model 940 Small animal; Kopf instruments, Tujunga, CA, USA) for the intrastriatal injection of sterile bacterial collagenase type VII-S (collagenase from *Clostridium histolyticum* Type VII-S; Sigma-Aldrich, Saint Louis, MO, USA). Using a microinjection pump (Model UMP3T-A; Kopf Instruments, Tujunga, CA, USA) and a NanoFil 26 G syringe (World Precision Instruments; Sarasota, FL, USA), 0.06 U of collagenase in 0.4 µL sterile saline was injected into the right striatum at a rate of 200 nL/min. The coordinates from Bregma for the stereotaxic injection were 0.5 mm anterior, 1.7 mm lateral, and 3.0 mm ventral. After the infusion and before syringe withdrawal, the needle was left in place for 10 min and the isoflurane was decreased to 1%. The syringe was removed at a rate of 1 mm/min in order to avoid retrograde flow, and the cranial burr hole was sealed with bone wax (B.Braun Vertcare; Rubí, Spain). The animals of the sham group received the same surgical procedure and were injected with 0.4 µL vehicle.

Once the common iron-related protein response in ischemic (n = 12) and hemorrhagic (n = 7) stroke models was established, the effect of hATf was tested in experimental ICH. The mice were randomly assigned to the groups ICH-vehicle (sterile saline) and ICH-human apotransferrin (hATf; Sigma-Aldrich, Saint Louis, MO, USA) at the dose of 230 mg/kg (n = 8 in each group). Exogenous hATf was used to be able to distinguish it from endogenous mouse ATf. Forty minutes after collagenase administration, vehicle or hATf was administered intravenously.

### 2.4. Tape Removal Test

Neurobehavioral performance was tested in daily training sessions in the 3 days prior to the ICH induction, and daily for 3 days after ICH induction to assess proprioception, asymmetry, and sensorimotor impairment through the adhesive tape removal test. Briefly, a round 6 mm diameter tape was stuck in the hand palm of the mouse in the forelimb contralateral forelimb to the hemorrhagic hemisphere. Both the time the mouse spent to notice/detect and to actively remove the tape were measured in each session. The mice received training in the test before ICH induction to avoid any stress derived from the novelty of the handling associated with the test performance.

### 2.5. Tail Bleeding Test

Nine male C57BL/6J mice were used to test a putative effect of exogenously administered hATf on coagulation (vehicle n = 5, hATf n = 4). Ten minutes after an i.v. administration of sterile saline or 230 mg/kg hATf, the tip of the tail was cut, the tail was submerged in physiological saline at 37 °C to ease bleeding, and the time elapsed until the tail stopped bleeding was measured.

### 2.6. Blood and Brain Sampling and Processing

Blood samples were obtained using EDTA-K or lithium heparin tubes (Microvette CB 300 EDTA or Microvette CB 300 lithium heparin; Sarstedt, Nümbrecht, Germany) before surgery, after the i.v. injection, and 24 h after the ICH induction. The plasma obtained after centrifugation was stored at −20 °C.

Three days after the treatments, the mice were euthanized via cervical dislocation, and the brains were quickly obtained and cut into 2 mm thick slices using a coronal mouse brain slicer matrix. Then, the slices were photographed on both sides. For Western blot and qPCR, the third fronto-caudal brain slice, the one showing the highest level of infarct or hematoma in the ischemic or hemorrhagic stroke models, respectively, was divided and saved separately into ipsilateral and contralateral hemispheres, flash-frozen in liquid N_2_, and stored at −80 °C. Further, the samples were lyophilized (Freeze Dryer; B.Braun Biotech; Melsungen, Germany) following Aliena-Valero et al.’s protocol [22] and stored at −80 °C until analysis. This way, we obtained a bias-free selection of the tissue for both WB and qPCR analyses. We chose this conservative procedure, despite it potentially underestimating the molecular changes in the ipsilateral hemisphere due to it also containing healthy tissue, as it allows for an objective standardization of the tissue collected for analyses.

For immunohistochemistry, the second rostro-caudal 2 mm thick slice obtained from the brain slicer matrix was preserved and stored at −80 °C. The most caudal side of this 2 mm slice, adjacent to the tissue used for qPCR and Western blot, was sliced using a cryostat into 15 µm thick slices to be used to detect 4-HNE and NeuN.

### 2.7. Assessment of the Ischemic Infarct Volume in the IS Model, Hemorrhage Volume, and Brain Hemoglobin and Heme Concentration in the ICH Model

The infarct volume and hemorrhage volume were quantified 72 h after treatments by measuring the 2,3,5-triphenyltetrazolium chloride (TTC)-unstained area or the hematoma area, respectively, in 2 mm thick coronal slices using ImageJ v1.53 (Wayne Rasband; NIH; Bethesda, MD, USA) and calculated as previously reported [23].

The brain hemoglobin and heme levels were measured using colorimetric detection assay kits (QuantiChrom Hemoglobin Assay Kit and QuantiChrom Heme Assay Kit; BioAssay Systems; Hayward, CA, USA) following the manufacturers’ instructions.

### 2.8. RT-qPCR

A miRNeasy Tissue/Cells Advanced Mini Kit (Qiagen; Hilden, Germany) was used for RNA isolation. Briefly, lyophilized brain tissue samples (ipsi and contralateral hemispheres separately) were resuspended in the supplied lysis buffer with 0.01% β-mercaptoethanol and homogenized by passing the lysate through a 21 G needle at least 5 times in order to isolate the RNA. The RNA was finally recovered in RNase-free water and its concentration and purity were assessed using a NanoDrop ND-1000 Spectrophotometer (NanoDrop; Thermo Fisher Scientific; Waltham, MA, USA). The same amount of RNA was used for the reverse transcription of each sample following QuantiTect Reverse Transcription Kit (Qiagen; Hilden, Germany) instructions. The samples were first incubated with a gDNA elimination reaction for 2 min at 42 °C and immediately placed on ice. The reverse transcription reaction was performed at 42 °C for 15 min, followed by inactivation at 95 °C for 3 min. A no-reverse transcription control was also prepared by replacing the reverse transcriptase with water. The obtained cDNA was then mixed with LightCycler 480 SYBR Green I Master (Roche Applied Science; Penzberg, Germany) and PrimeTime^®^ qPCR Primers for different genes (Integrated DNA Technologies; Coralville, IA, USA). Real-time PCR was performed in a Real-Time PCR Roche LightCycler 480 I (Roche Applied Science; Penzberg, Germany). The reaction was initiated using a pre-incubation step of 5 min at 95 °C, followed by 45 cycles of amplification at 95 °C for 10 s, 60 °C for 10 s, and 72 °C for 10 s. Triplicates of the samples and melting curve analysis were also performed. A no-template control was included, replacing the sample cDNA with RNase-free water, as well as a positive control using Universal Mouse Reference RNA (Invitrogen; Waltham, MA, USA). Data analysis was performed using the ΔΔCt method and GAPDH was utilized as a housekeeping gene. Commercial proprietary predesigned qPCR forward and reverse primers obtained from IDT (Integrated DNA Technologies; Coralville, IA, USA) were used (Table S1).

### 2.9. Western Blot (WB)

Lyophilized brain samples were reconstituted in the same lysis buffer used for the hemoglobin and heme quantification. The protein content of the reconstituted samples was determined using a Pierce™ BCA Protein Assay Kit (Thermo Fisher Scientific; Waltham, MA, USA). Then, 30 µg of total protein from the brain was loaded in Precast NuPAGE Midi 10% Bris-Tris (Thermo Fisher Scientific; Waltham, MA, USA). A molecular weight marker (Precision Plus Protein^TM^ Standards; Bio-Rad; Hercules, CA, USA) was included in the gels and GAPDH was used as a tissue sample loading control.

For the TSAT assessment, 0.15 µL of plasma sample was loaded in Precast 6% TBE urea gels (U-PAGE) (Thermo Fisher Scientific; Waltham, MA, USA). These gels included human apotransferrin and human holotransferrin (hHTf) (Sigma-Aldrich; Saint Louis, MO, USA), as well as in-lab prepared mouse ATf and HTf standards, which were prepared following the methods described in Byrne S et al. and Nagaoka MH et al. [24,25].

PVDF-LF membranes (Millipore; Burlington, VT, USA) were used for the electroblotting of the gels, which were blocked with Intercept**^®^** Blocking Buffer (Li-COR Biosciences; Lincoln, NE, USA) for 1 h. Then, the membranes were incubated overnight at 4 °C with the specific primary antibodies. After that, they were incubated with NIR-conjugated secondary antibodies. The bands were measured using an Odyssey Imaging system and Image Studio Lite software v5.2 (Li-COR Biosciences; Lincoln, NE, USA). The values were measured in terms of mean integrated density (ID) in arbitrary units ((mean optical density of pixels of the specific signal) − (mean background optical density)). Then, for each protein, 1.00 was assigned to the ID of the contralateral brain hemisphere and the ID of the ipsilateral hemisphere was expressed in terms of the fold-change of that of the contralateral hemisphere.

### 2.10. Determination of 4-Hydroxynonenal (4-HNE) Using Immunohistochemistry (IHC)

In this stage, 15 µm thick brain slices from frozen tissue were obtained using a cryostat (Leica CM1950; Leica Biosystems; Deer Park, TX, USA) on poly-L-lysine-coated glass slides and fixed in 4% paraformaldehyde. They were exposed to antigen retrieval (90–95° in 0.01 mol/L citrated buffer, pH 6.0, for 20 min), incubated overnight with the primary antibodies at 4 °C, and then with the secondary antibodies and Hoechst (33342; Thermo Fisher Scientific; Waltham, MA, USA). Fluoromount (Sigma-Aldrich F4680; Saint Louis, MO, USA) was used as the mounting medium. Images were obtained using an AxioOberver Z1 microscope (Carl Zeiss; Oberkochen, Germany) in the Microscopy Platform Core Facility at the IGTP and analyzed using ImageJ. NeuN immunohistochemistry was macroscopically imaged on an Odyssey Imaging System. In brief, the 15 µm thick brain slice that preceded the slice used for microscopy studies was subsequently incubated with anti-NeuN antibody and with an NIR-conjugated secondary antibody for Odyssey imaging. Due to the loss of neurons, almost no NeuN staining was observed within the hematoma in the whole picture of the Odyssey-imaged brain slice. The cortical areas neighboring the hematoma had neurons potentially exposed to ICH-produced toxic compounds. Under the microscope, the areas devoid of NeuN, which are hematoma, together with the anatomical brain references easily identified in the slice under the microscope (e.g., corpus callosum, ventricles), enabled us to identify periinfarct areas of interest in the nearby cortex. Several different series of images were obtained and 4-HNE was detected and quantified in NeuN-positive neurons in the areas of interest.

### 2.11. TSAT Assessment

Basal TSAT levels were determined before the onset of the experimental stroke procedures to check that mice had TSAT levels similar to those in the human population (<40%), and 24, 48, and 72 h after ICH induction.

The U-PAGE gels mentioned above separate transferrin into ATf (Tf devoid of iron), monoferric Tf (mFe, an Fe atom in the C-terminal region or in the N-terminal region) and diferric Tf (diFe, holotransferrin, HTf), giving different bands according these Tf isoforms. The % TSAT was calculated using the measurement of the bands from the U-PAGE gels and the TSAT formula previously used by us and others [3,26]:

$$TSAT\left(\%\right)=\left(\frac{1}{2}\times mFe\cdot Tf+diFe\cdot Tf\right)\times 100/(ATf+mFe\cdot Tf+diFe\cdot Tf)$$

### 2.12. Antibodies

The primary and secondary antibodies used for WB and IHC (see Table S2) were from Thermo Fisher Scientific (Waltham, MA, USA), Abcam (Cambridge, UK), Novus Biologicals (Centennial, CO, USA), Santa Cruz Biotechnology (Dallas, TX, USA), Green Mountain Antibodies (Burlington, VT, USA), Cappel, ICN Pharmaceuticals (Costa Mesa, CA, USA), and Sigma-Aldrich (Saint Louis, MO, USA), Li-COR Bioscience (Lincoln, NE, USA) and Thermo Fisher Scientific (Waltham, MA, USA).

### 2.13. Statistics

Original or log-transformed data were analyzed using GraphPad Prism 9. A paired or unpaired Student’s *t*-test or independent or repeated-measures one-way ANOVA followed by the multiple comparisons Tukey’s test were used, as appropriate. Statistical significance was considered at *p* < 0.05. Data are presented as the mean and SEM.

## 3. Results

### 3.1. Gross Differences and Commonalities between Ischemic and Hemorrhagic Stroke Models

**Figure 1:**
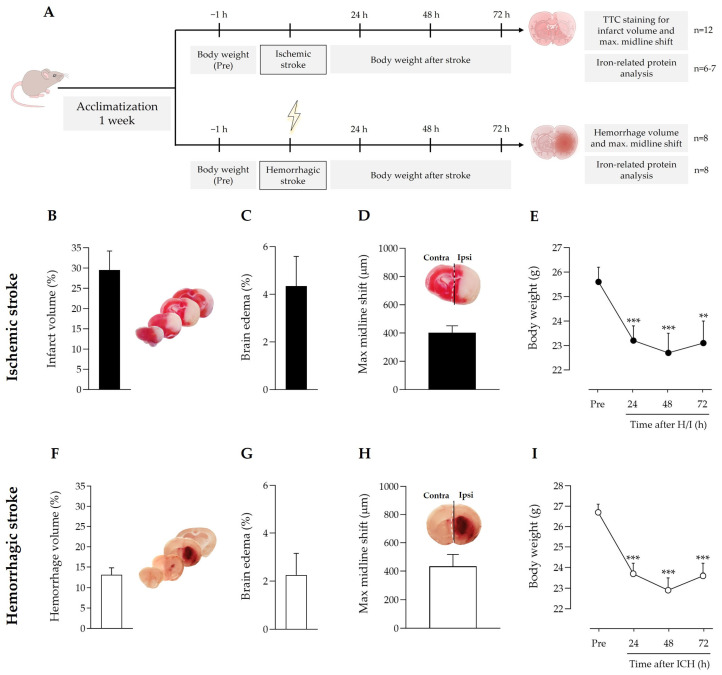
(**A**) Timeline scheme of the experimental groups and procedures. Effect of (**B**–**E**) ischemic stroke and (**F**–**I**) intracerebral hemorrhage on (**B**,**F**) infarct or hemorrhage volume, (**C**,**G**) edema, (**D**,**H**) stroke-induced maximum hemispheric midline shift (broken white line) vs. the theoretical midline (solid black line), and (**E**,**I**) body weight loss; ** *p* < 0.01 and *** *p* < 0.005 vs. pre-stroke (repeated measures one-way ANOVA and Tukey’s test). Data are represented as the mean and SEM. Contra: contralateral; ipsi: ipsilateral. Representative images of ischemic regions (regions in white in the brain slices in (**B**) and hemorrhagic regions (reddish areas in the brain slices in (**F**) in coronal brain slices are shown and were used to quantify ischemic infarct volume and hemorrhagic stroke volume, respectively.

The nature, extent, and location of the brain regions affected by stroke differ between the two stroke models. There is a large corticostriatal infarct in ischemia, and smaller, mainly striatal, hemorrhage in ICH (Figure 1B,F). Despite this, body weight loss followed the same pattern in both ischemic and ICH mice (Figure 1E,I), and the same applied for edema (Figure 1C,G) and the cerebral midline shift induced by the ipsilateral hemisphere enlargement (Figure 1D,H).

### 3.2. Ischemic and Hemorrhagic Stroke Share a Common Profile of Iron-Binding, Ferroptosis-Related Proteins in the Ipsilateral Brain Hemisphere 72 h after Stroke Induction

**Figure 2:**
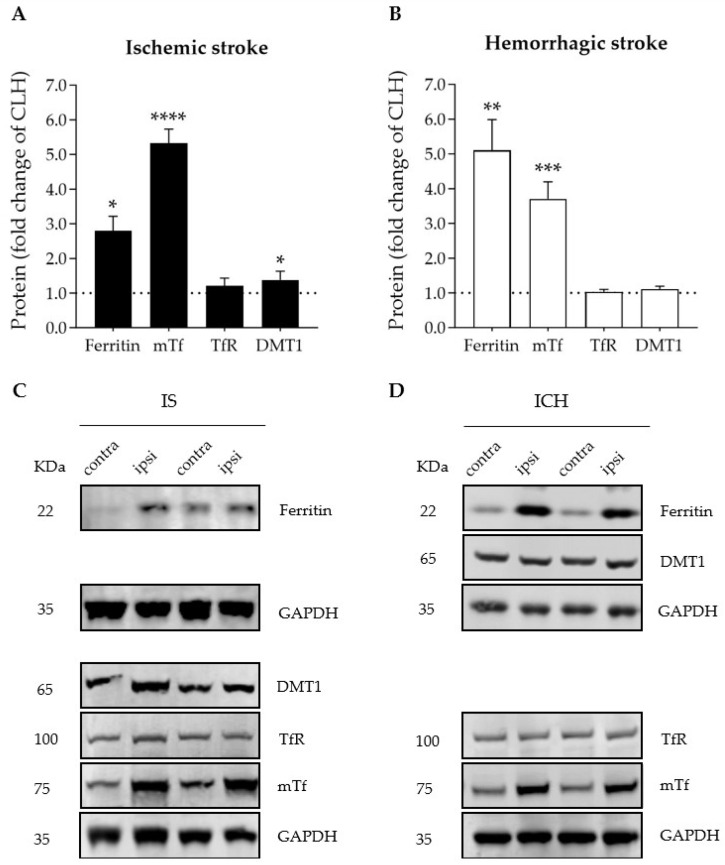
Graphs depicting the effect of (**A**) ischemic stroke and (**B**) intracerebral hemorrhage on ipsilateral brain levels of iron- and ferroptosis-related proteins 72 h after stroke onset, expressed as fold change of those of the contralateral hemisphere (CLH, mean represented by the dotted line at y = 1.0); * *p* < 0.05, ** *p* < 0.01, *** *p* < 0.005, and **** *p* < 0.0001 vs. contralateral hemisphere (paired *t* test), n = 6–7. Mean and SEM are shown. (**C**,**D**) Representative WB of the bands of the proteins of interest in the contralateral (contra) and ipsilateral (ipsi) hemispheres of (**C**) ischemic stroke (IS) and (**D**) intracerebral hemorrhage (ICH). DMT1: divalent metal transporter 1; GAPDH: glyceraldehyde-3-phosphate dehydrogenase; mTf: mouse transferrin; TfR: transferrin receptor. GAPDH was used as the housekeeping protein and is shown in the figure as the bottom row in each one of the four representative membranes depicted (no more than four antibodies were tested in the same membrane).

Importantly, compared with the contralateral brain hemisphere, both ischemic and hemorrhagic stroke increased the protein levels of iron-storage ferritin and the iron-binding and carrier transferrin in the ipsilateral hemisphere (Figure 2A,B). A modest increase in the levels of divalent metal transporter 1 (DMT1), which imports ferrous iron to the cytosol, was observed in the ipsilateral hemisphere of the ischemic stroke mice only (Figure 2A); no changes were observed in the levels of transferrin receptor (TfR) (Figure 2A,B), which plays a pivotal role as the main gate for iron entry into the endothelial cells of the brain capillaries and into neurons; also, TfR has recently been identified as a ferroptosis biomarker [27].

### 3.3. Early Peripheral Administration of hATf into the Bloodstream after ICH Induction Does Not Reduce Parenchymal Hemorrhage

The common iron-binding, ferroptosis-related protein signature we observed in both the ischemic and the hemorrhagic models above, together with the fact that our group has previously demonstrated that ATf exerts neuroprotection in ischemic stroke models in rats [3], provided the rationale to study the neuroprotective potential of iron-devoid ATf in the ICH mouse model. We administered i.v. human ATf (hATf) to be able to distinguish it from endogenous mouse ATf in the WB, using the appropriate gel conditions and the specific species antibodies already tested in our lab [3].

**Figure 3:**
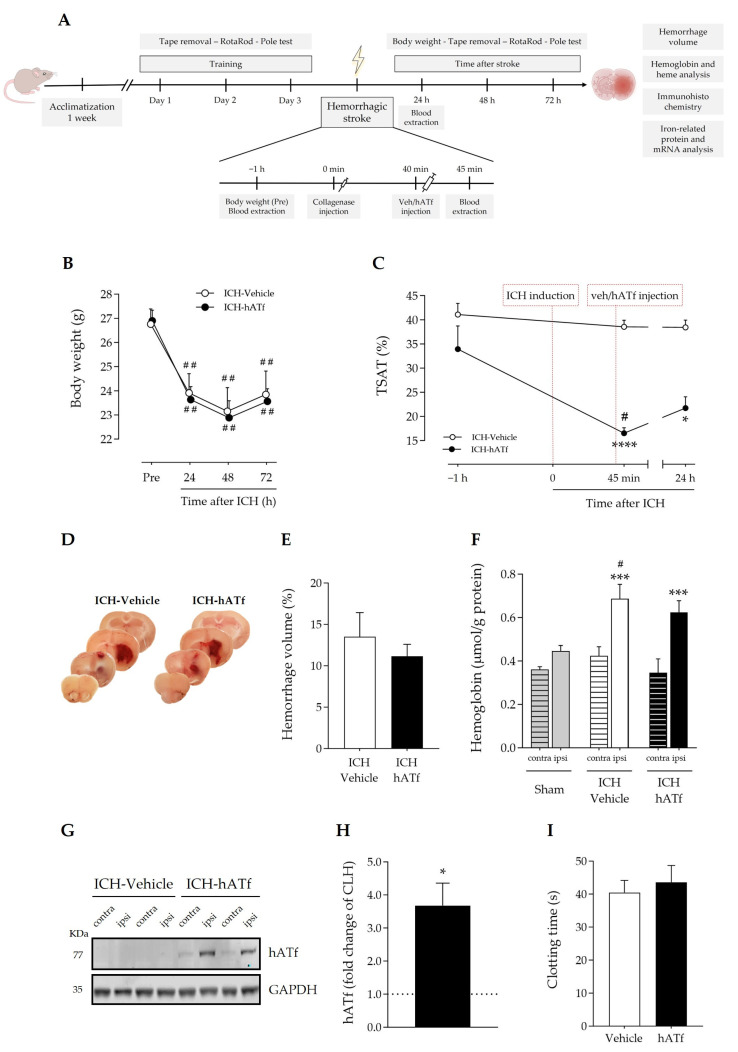
(**A**) Timeline scheme of the experimental groups and procedures to determine the effect of hATf administration in ICH mice. (**B**) Body weight loss; ## *p* < 0.01 vs. respective Pre (repeated-measures (RM) one-way ANOVA and Tukey’s test). (**C**) Time course of %TSAT before and after ICH induction and i.v. administration of vehicle or hATf; * *p* < 0.05 and **** *p* < 0.0001 vs. pre-administration (−1 h) measure (RM one-way ANOVA and Tukey’s test), # *p* < 0.05 vs. ICH-Vehicle (*t* test). (**D**) Representative images of hemorrhage in coronal brain slices and (**E**) quantification of the hemorrhage volume. (**F**) Hemoglobin levels in the contralateral (contra) and the ipsilateral (ipsi) hemispheres of ICH mice given vehicle or hATf; a group of sham mice was included; *** *p* < 0.005 vs. respective contra (paired t test), # *p* < 0.05 vs. Sham-ipsi (*t* test). (**G**) Representative WB showing hATf levels in the contralateral (contra) and the ipsilateral (ipsi) hemispheres of ICH mice administered vehicle or hATf and (**H**) its quantification; * *p* < 0.05 versus contralateral (CLH, mean represented by the dotted line at y = 1.0; paired t test). (**I**) Tail bleed clotting time in mice given vehicle or hATf. Mean and SEM are shown.

Figure 3C shows that ICH itself did not alter blood transferrin saturation with iron, also known as TSAT, which remained at around 40% before, and 45 min and 24 h after ICH induction. By contrast, hATf reduced TSAT to approximately half the pre-ICH values 5 min after administration (hence, 45 min after ICH induction), and TSAT remained low in these animals 24 h later. These observations are very similar to those we reported in ischemic stroke models in rats [3].

On the other hand, blood extravasation in the ipsilateral hemisphere in both ICH-vehicle and ICH-hATf mice was evident and of comparable magnitude in the gross histology (Figure 3D), and no significant difference was observed in the hemorrhagic volume 72 h after ICH onset (Figure 3E), or in terms of hemoglobin (Figure 3F) or heme levels (Figure S2). In the WB, hATf was not detected in the cerebral hemisphere of the mice administered the vehicle, as expected, and the hATf signal was very low in the contralateral hemisphere and high in the ipsilateral hemisphere of the mice given hATf (Figure 3G,H). Also, hATf treatment altered neither body weight loss (Figure 3B) nor the tail bleed clotting time (Figure 3I).

### 3.4. hATf Reduces ICH-Induced Sensorimotor Impairment in the Adhesive Tape Detection and Removal Test

**Figure 4:**
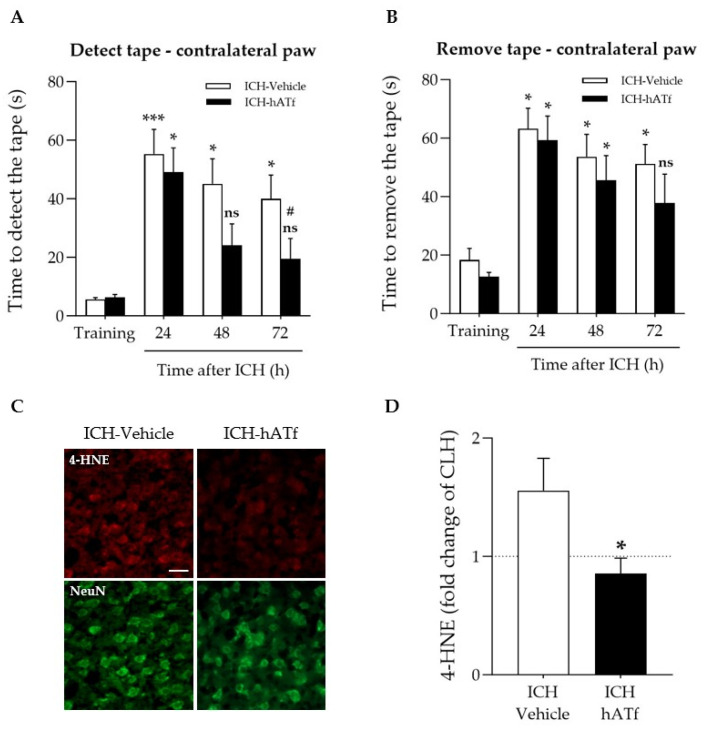
Effect of ICH and hATf on the neurobehavioral performance and brain 4-HNE levels. (**A**,**B**) Graphs depicting the hATf-induced improvement in the adhesive tape detection/removal test of the contralateral paw showing (**A**) time to detect and (**B**) the time to remove the tape at 24, 48, and 72 h post-ICH. * *p* < 0.05 and *** *p* < 0.005 vs. respective training; ns means non-significant versus training (mice performance before being exposed to ICH), # *p* < 0.05 vs. respective 24 h (repeated-measures one-way ANOVA and Tukey’s test). (**C**) Representative immunohistofluorescence images of 4-hydroxynonenal (4-HNE) and NeuN in the perihematomal cortical area of the ipsilateral hemisphere of ICH-Vehicle and ICH-hATf mice; scale bar, 20 µm. (**D**) Graph depicting 4-HNE levels in neurons in the perihematomal cortical area in fold change of mirror areas of the CLH (mean represented by the dotted line at y = 1.0). * *p* < 0.05 vs. vehicle. Mean and SEM are shown.

As expected, ICH considerably increased the time the mice took to detect (Figure 4A) and to remove (Figure 4B) the tape from the contralateral paw compared to the training session prior to ICH induction. The administration of hATf after ICH induction progressively reduced the ICH-induced sensorimotor impairment, reducing the time required to detect the tape at 48 and 72 h and to remove the tape at 72 h (ns: non-significant) compared with their performance before ICH (training). ICH mice treated with hATf also showed significant improvement at 72 h when compared with their post-ICH performance at 24 h (# *p* < 0.05). Vehicle-treated, but not ATf-treated, ICH animals had increased 4-HNE levels in the perihematomal tissue of the ipsilateral hemisphere, as determined by immunohistochemistry (Figure 4C,D).

### 3.5. Effect of hATf on Iron Storage/Transport-Related mRNA and Protein Levels in ICH Mice

**Figure 5:**
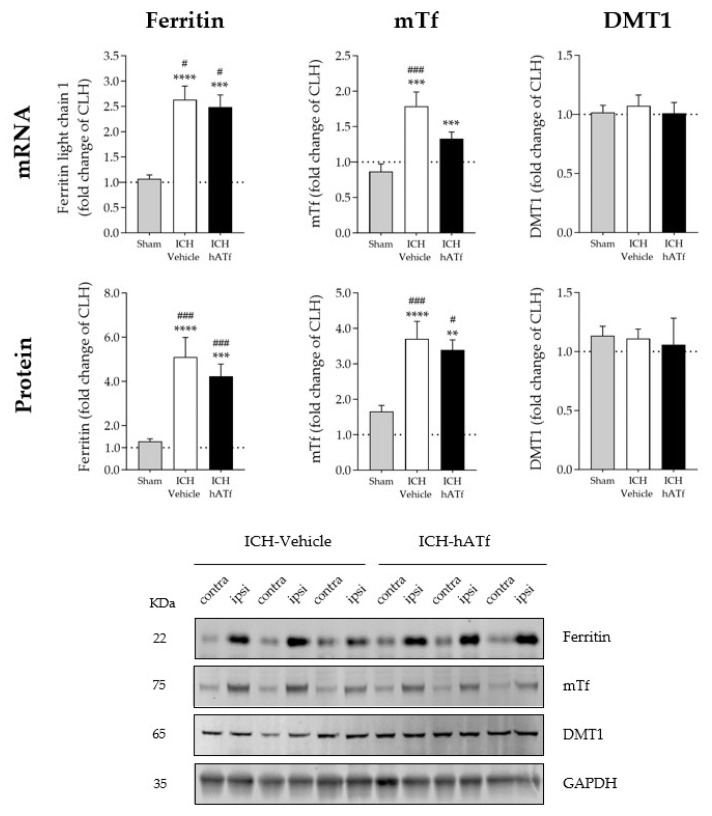
Graphs showing the effect of ICH and hATf on mRNA (**top**) and protein (**bottom**) levels of ferritin, endogenous mouse transferrin (mTf), and divalent metal transporter 1 (DMT1) in the ipsilateral cerebral hemisphere 72 h after ICH induction, expressed as fold change of the contralateral hemisphere (CLH, mean represented by a dotted line at y = 1.0). ** *p* < 0.01, *** *p* < 0.005, and **** *p* < 0.0001 vs. CLH (paired *t* test); # *p* < 0.05, and ### *p* < 0.005 vs. sham (one-way ANOVA and Tukey’s test). Mean and SEM are shown. Bottom panel: representative WB of the contralateral (contra) and ipsilateral (ipsi) hemispheres; glyceraldehyde-3-phosphate dehydrogenase (GAPDH) was used as the housekeeping protein.

As shown in Figure 5, ICH plus either vehicle or hATf increased ferritin and endogenous transferrin mRNA and protein levels in the ipsilateral hemisphere compared with the respective contralateral hemisphere (CLH) and with sham mice. However, the increases in mouse transferrin mRNA and protein levels were of less magnitude in the hATf-treated mice than they were in the vehicle mice.

### 3.6. Effects of hATf on Classical Key Ferroptosis mRNA and Protein Levels in ICH Mice

**Figure 6:**
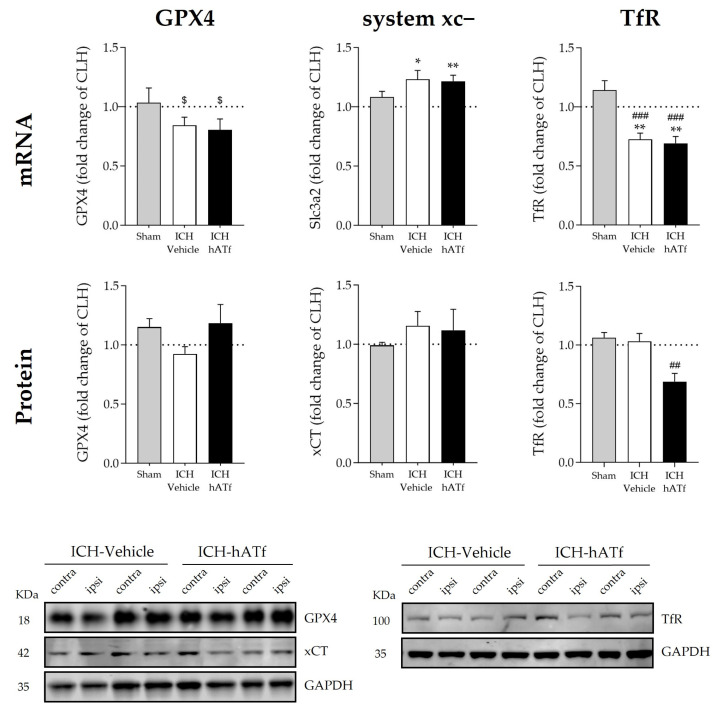
Graphs showing the effect of ICH and hATf on mRNA (**top**) and protein (**bottom**) levels of glutathione peroxidase 4 (GPX4), Tf receptor (TfR), and the cysteine/glutamate antiporter system xc^−^ in the ipsilateral cerebral hemisphere 72 h after ICH induction, expressed as fold change of the contralateral hemisphere (CLH, mean represented by a dotted line at y = 1.0). * *p* < 0.05 and ** *p* < 0.01 vs. contralateral hemisphere (paired *t* test), ## *p* < 0.01 vs. sham and ICH-vehicle (TfR protein), ### *p* < 0.005 vs. sham (TfR mRNA) (one-way ANOVA and Tukey’s test), $ 0.06 < *p* < 0.07 vs. contralateral hemisphere (GPX4 mRNA) (paired *t* test). Mean and SEM are shown. Representative WB of GPX4, xCT, and TfR in the contralateral (contra) and ipsilateral (ipsi) hemispheres of vehicle and hATf-treated mice are shown; glyceraldehyde-3-phosphate dehydrogenase (GAPDH) was used as the housekeeping protein.

No statistically significant effects of ICH were found in glutathione peroxidase 4 (GPX4) mRNA and protein levels. Nonetheless, GPX4 mRNA levels showed a trend of being lower in the ipsilateral hemisphere of the ICH mice when compared with sham animals (*p* = 0.0624 in ICH-vehicle and *p* = 0.0681 in ICH-hATf mice) (Figure 6). ICH increased system xc- gene expression (Slc3a2 mRNA) irrespective of the treatment; at the protein level (xCT), it did not reach statistical significance (Figure 6). ICH induced a similar decrease in TfR mRNA levels in both vehicle and hATf mice, but only hATf provoked a concomitant decrease in TfR at the protein level.

### 3.7. Effect of hATf on the mRNA Expression of Newly Reported Key Ferroptosis Players AIFM2/FSP1 and PCBP2 in ICH Mice

**Figure 7:**
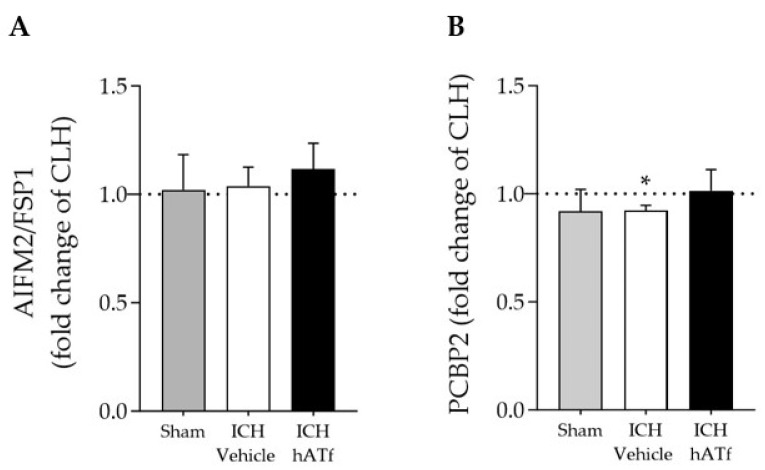
Effect of ICH and hATf on mRNA levels of (**A**) ferroptosis-related apoptosis-inducing factor 2 (AIFM2/FSP1) and (**B**) the iron chaperone poly(rC)-binding protein 2 (PCBP2) in the ipsilateral cerebral hemisphere 72 h after ICH induction, expressed as fold change of the contralateral hemisphere (CLH, mean represented by a dotted line at y = 1.0). * *p* < 0.05 vs. contralateral hemisphere (paired *t* test). Mean and SEM are shown.

Neither ICH nor hATf changed the mRNA expression of apoptosis-inducing factor 2 (AIFM2, also known as ferroptosis suppressor protein 1, FSP1), a newly identified ferroptosis player (Figure 7A). We found a slight decrease in the mRNA levels of the cytosolic iron chaperone poly (rC)-binding protein 2 (PCBP2) in the ICH-vehicle animals that was no longer observed in the ICH-hATf-treated animals (Figure 7B).

### 3.8. hATf Reduces ICH-Induced Caspase 2 Activation

**Figure 8:**
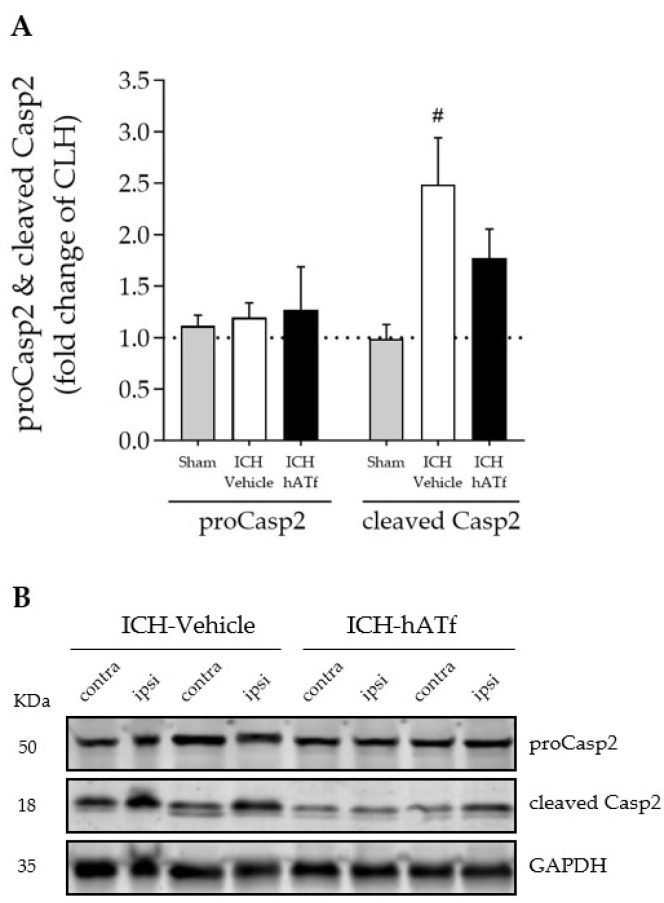
(**A**) Effect of ICH and hATf on brain caspase-2 (proCasp2) activation by cleavage into the p18 form (cleaved p18) in the ipsilateral cerebral hemisphere 72 h after ICH induction, expressed as fold change of the contralateral hemisphere (CLH, mean represented by a dotted line at y = 1.0). (**B**) Representative WB showing proCasp2 and cleaved Casp2 in the contralateral and ipsilateral hemispheres of vehicle- and hATf-treated mice. # *p* < 0.05 vs. respective sham (one-way ANOVA and Tukey’s test). Mean and SEM are shown.

ICH increased the levels of cleaved Casp2 p18 form, which was partly prevented by the administration of hATf (Figure 8A).

## 4. Discussion

Here we demonstrate, in an in vivo collagenase-induced ICH model in mice, that a single intravenous administration of hATf shortly after stroke induction is not only safe, but shows efficacy in reducing the sensorimotor impairment of the affected forepaw (Figure 4). The results of the study also suggest that the benefits of the hATf treatment might be exerted through an antiferroptotic mechanism independent of the canonical inhibitor of the ferroptosis system xc-/GPX4 axis.

Our results show that both stroke models, ischemic and hemorrhagic, provoked similar body weight loss, and swelling and a shift of the ipsilateral brain hemisphere beyond the sagittal midline (Figure 1). We also observed similar effects in both stroke models (a 2.8- to 5.3-fold increase) in the levels of the main iron storage and transport proteins, ferritin and endogenous mouse Tf, respectively, in the ipsilateral hemisphere with a minor effect, or none at all, of ICH on the levels of the main molecular gates for iron passage into the cytosol: TfR at the cell membrane and DMT1 (also known as SLC11A2, DCT1, or Nramp2) as the lysosome-to-cytosol gate (Figure 2). This points to a common picture of iron dyshomeostasis with the need to safely store excess cellular iron in the intracellular iron-storage protein ferritin in stroke arising from both etiologies, which is in agreement with previous literature on the issue [3,11,28,29,30,31]. Therefore, molecules with the ability to prevent iron overload in neurons would be good putative candidates to alleviate stroke-induced neurodegeneration, as has also been previously reported for deferoxamine in ICH or ischemic stroke patients [4,32]. Our group has also already reported the protective effect for hATf in ischemic stroke models [3].

The clearance and iron status in the blood of the hATf administered in ICH mice is similar to that previously reported in ischemic stroke in rats [3]. Thus, hATf administration immediately reduced plasma TSAT compared to mice given the vehicle (as assessed 5 min later), an effect that lasted for at least 24 h post-ICH (Figure 3C). Also, exogenous hATf reached and remained in the brain, as assessed at 72 h, at higher levels in the ipsilateral than in the contralateral hemisphere (Figure 3G,H). Of note, the presence of hATf in the brain parenchyma is associated with a reduction in the neuronal levels of 4-HNE in the ICH-affected perihematomal areas of the hemisphere (Figure 4C,D). Interestingly, previous work demonstrates that the addition of hATf in vitro to the extracellular medium of excitotoxically challenged neurons in culture, being equivalent to lowering TSAT in vivo, decreases neuronal oxidative stress [3], whereas the addition of iron-loaded transferrin (holotransferrin, HTf) to ischemic or excitotoxically challenged neurons increases ROS production and harmful neuronal iron uptake [3]. As for the neurological outcome, we found that treatment with hATf induced better performance in the sensory aspects of the adhesive tape removal test when compared to placebo mice. The hATf-treated ICH mice regained sensorimotor skills in this test, which measured front paw tasks (Figure 4A), although they did not regain general coordination (Figure S1). There was no statistical difference in the contralateral paw detection performance of hATf-treated mice at 48 and 72 h, nor in the time taken to remove the tape 72 h after the ICH onset compared to the results from pre-ICH training sessions. This was different to mice given the vehicle, and, thus, hATf-treated mice show an improvement in their post-ICH performance in a complex task requiring sensory, motor, and coordination skills.

While searching for other underlying mechanisms through which hATf provides sensorimotor improvement in ICH mice, we investigated the effects of hATf administration on: (1) the size of the brain hematoma and changes in blood coagulation, and (2) iron- and ferroptosis-related molecules relevant to stroke.

Regarding the size of the hematoma, despite hATf reaching the hemorrhagic brain hemisphere, it did not reduce the ICH-induced hemorrhage volume, the edema, or the hemoglobin and heme concentration in the ipsilateral hemisphere when administered in a relevant time window (40 min post-collagenase) (Figure 3 and Figure S2). In addition, we did not find any effect of hATf treatment on the clotting time assessed in the tail-bleeding time test in the systemic circulation in healthy mice (Figure 3I). Moreover, there was no any significant alteration in the parameters of the coagulation cascade when hATf was incubated in vitro with freshly obtained human blood in experimental in vitro conditions that mimic as closely as possible (1) the concentration of hATf in blood, and (2) the blood dilution produced when adding the treatment volume in the mice blood (Figure S3). Of note, we tested these parameters and obtained these negative results in two species, in physiological conditions, and in acute scenarios, all of which presented as very relevant for stroke. However, other studies tested the effects of transferrin on coagulation in other conditions, reporting either procoagulant effects, e.g., chronically elevated transferrin levels or blood processed much further than we did [33,34] or, in contrast, higher transferrin associated with protection from thrombosis [35].

Regarding the iron- and classical ferroptosis-related molecules relevant to stroke, we found no significant effect of ICH, suggesting that a physiological attempt to maintain normal GPX4 protein levels and to provide enough supply of cystine, through system xc, cysteine, and, ultimately, the GPX4 cofactor GSH to reduce the ICH-induced lipid peroxidation is taking place. Neither ICH nor hATf altered the protein levels of GPX4 or xCT (SLC7A11), although GPX4 mRNA levels showed a trend of being reduced (0.06 < *p* < 0.07) and the mRNA levels of the heavy-chain subunit of the system xc- (Slc3a2) were increased in the ipsilateral hemisphere of the ICH mice, irrespective of whether they had received vehicle or hATf (Figure 6). Neither ICH nor hATf altered the mRNA levels of ferroptosis suppressor protein 1 (FSP1 or AIFM2) (Figure 7), which stands for another key ferroptosis axis, the FSP1/CoQ10/NAD(P)H pathway, which has only received attention very recently [36,37]. Interestingly, ICH reduced the mRNA levels of the cytosolic iron chaperone PCBP2; this reduction was prevented by hATf treatment and is relevant, since knocking down PCBP2 has been recently reported to promote ferroptosis [38].

In addition, and of special interest regarding a putative anti-ferroptotic effect of hATf, ICH decreased TfR1 mRNA levels in both vehicle- and hATf-injected mice, whereas only the hATf-treated animals showed a concomitant reduction in TfR protein levels (Figure 6), which, as the main membrane cellular gate of iron entry into the cell, might be directly implicated in the benefit induced by hATf treatment. TfR1 abundance, especially at the cell membrane, is a known hallmark promotor of ferroptosis [27,39]. This is in line with a recent report that shows that the knockdown of TfR1 inhibits ischemia/reperfusion-induced ferroptosis in the rat heart through the inactivation of the p53/TfR1 pathway [40]. Another recent study reports that ferroptosis in the hippocampus and cognitive dysfunction induced by the i.c.v. injection of lipopolysaccharide are prevented by the downregulation/suppression of TfR1 expression [41,42]. Of note, the reduction in the TfR mRNA levels observed in ICH would be in accordance with the reported in vitro effect of hemin as an epigenetic negative regulatory factor upon transcription of the transferrin receptor gene [43].

The effect of ICH on the parenchymal levels of the key molecule involved in ferroptosis GPX4 is controversial, with some authors reporting no changes at 72 h [18,19], whereas others report reductions in mRNA and protein expression at 72 h [17,43,44,45]. Our results, which only show a trend of reduction in the mRNA levels, added to the disparities in the literature. It is plausible that the differences in the methodology of the few studies carried out to date on this issue may account for the discrepancies. With regard to this, we found large differences in the GPX4 levels present in young/undifferentiated neurons in culture compared to differentiated ones, with young neurons containing low levels of GPX4 being more susceptible to ferroptosis (Figure S4). Moreover, isoflurane has been reported to have either neuroprotective or neurotoxic effects [46], and it has been recently reported to reduce hippocampal GPX4 levels after long exposures (1.5% for more than 2h) [47]. In the present experiments, although no mice were exposed to more than 1 h isoflurane, we cannot rule out that an isoflurane-induced reduction in GPX4 levels in both hemispheres might have masked the ICH-induced effect in the ipsilateral hemisphere to some degree.

Wang et al. reported that the restoration of GPX4 and FSP1/AIFM2 levels through the early administration of the antioxidant dexpramipexole maintains white matter integrity and improves locomotion and motor coordination in the model of ICH induced by the injection of autologous blood in the striatum [48]. Our results here suggest that even when the two pathways mentioned are not significantly affected, iron-burden-dependent ferroptosis is occurring, and this could take place mainly through the p53/TfR1 pathway. This fits the recent understanding of ferroptosis as a flexible process under the regulation of many different functionally related molecules. The fact that hATf binding to the remaining TfR1 exerts a competitive effect on the iron-loaded transferrin binding and internalization through this receptor suggests that exogenous hATf plays a decisive role in preserving brain parenchyma and neuronal iron homeostasis. The recent identification of a novel lncRNA that constitutively represses p53 and apoptosis in cooperation with PCBP2 [49] provides a nexus between the p53/TfR1 pathway, PCBP2, and apoptosis, and strongly suggests that there is crosstalk between the pathways driving to apoptosis and to ferroptosis. Since, in vitro, PCBP2-specific siRNA-transfected neurons show a significant decrease in apoptosis following glutamate stimulation [50], we investigated the effect of ICH and hATf treatment on the activation of caspase-2, one of the upstream caspase effectors. We observed that the ICH-induced cleavage of procaspase-2 that renders an increase in active caspase-2 was abrogated by treatment with hATf (Figure 8). The fact that PCBP2 knockdown significantly increases the sensitivity to erastin-induced ferroptosis in cancer cells, whereas it reduces glutamate-induced apoptosis in neurons [38,50], points to a prominent dual antiferroptotic and proapoptotic role of PCBP2; the balance between the two processes probably depends on the cell type and its physiological status, with all of these together finally determining the cell fate.

The mechanisms that finally underlie the improvement in the sensorimotor abilities of ICH mice treated with hATf are likely to be multifactorial and related to its effect on ferroptosis players, such as TfR1 and PCBP2, and on the inactivation of caspase-2. In addition, the previously reported effects of ATf increasing the proliferation and maturation of remyelinating oligodendrocytes and on myelin deposition [51] might also contribute.

In summary, ATf administration is safe and provides sensorimotor improvement to mice exposed to experimental ICH. The present results, together with the benefit previously demonstrated for ATf in experimental ischemic stroke, allow the proposal of ATf as a pre-hospital frontline treatment that could be administered to patients very early on, even en route to an accurate in-hospital differential diagnostic.

## Data Availability

Data are available upon reasonable request.

## Supplementary Materials

The following supporting information can be downloaded at: https://www.mdpi.com/article/10.3390/antiox12111945/s1: Table S1: Commercial proprietary predesigned qPCR forward and reverse primers used; Table S2: Antibodies used for WB and immunohistochemistry (IHC); Figure S1: Effect of ICH and hATf on the neurobehavioral performance of mice in the pole test and the rotarod; Figure S2: Heme levels in the contralateral and the ipsilateral hemispheres of ICH mice given vehicle or hATf; Figure S3: Effect of human ATf and human HTf on human blood coagulation; Figure S4: Effect of erastin on neuronal viability in vitro and levels of GPX4 and xCT along neuronal maturation; Figure S5: Body weight of sham H/I and sham ICH mice over the course of the experimental period; Gels S1–S6: Original full WB gels.
