# Supplementary material for: Targeting Pro-Oxidant Iron with Exogenously Administered Apotransferrin Provides Benefits Associated with Changes in Crucial Cellular Iron Gate Protein TfR in a Model of Intracerebral Hemorrhagic Stroke in Mice
Source: Antioxidants (Basel). 2023 Oct 31;12(11):1945. doi: 10.3390/antiox12111945 (PMC10669272; doi:10.3390/antiox12111945)

Targeting Pro-Oxidant Iron with Exogenously Administered Apotransferrin Provides Benefits Associated with Changes in Crucial Cellular Iron Gate Protein TfR in a Model of Intracerebral Hemorrhagic Stroke in Mice

Alexia García-Serran [1], Jesús Ordoño [1], Núria DeGregorio-Rocasolano [1], Marc Melià-Sorolla [1], Karla Odendaal [1,2], Octavi Martí-Sistac [1,3,*] and Teresa Gasull [1,*]

Supplementary Materials

Neurobehavioral tests

In the pole test, the mouse was placed head up on the top of a pole (55 cm high and 1 cm diameter) and the time that the mice required to turn around and go down was measured. In the rotarod test the mice was placed on an accelerating rod (Rota Rod LE8200; Harvard Apparatus, Holliston, USA), and the amount of time the mouse spent on the accelerating rod before it fell down was measured. In each session (at 24, 48 or 72 h post ICH), the test was repeated thrice and mean value was depicted. Mice received a 2-3-day training in the test before ICH induction.

Coagulation tests of human blood

Coagulation tests in human blood were performed by the Hematology service of the Germans Trias i Pujol Hospital using 1.8 mL BD™ Vacutainer™ Citrate Tubes (Thermo Fisher Scientific; Waltham, USA). A total amount of 129 µL of hATf (Sigma-Aldrich, Saint Louis, USA) or hHTf (Sigma-Aldrich, Saint Louis, USA) at a concentration of 50 mg/mL was placed in the tubes. Physiological saline serum was used as a control. For the test in vitro in human blood samples, we performed the experiment as close as possible to the in vivo conditions by adding the same amount, concentration, and relative volume of hATf to the freshly extracted human blood plasma samples that ought to be in the blood of mice minutes/hours after the treatment.

Neuronal cell culture

Neuronal cultures were obtained from rat-fetuses (Sprague-Dawley rats from Envigo/Harlan)-cortical neurons and prepared as previously described [3]. At 3 and 11 days-in-vitro (DIV), cultures were exposed to either Erastin 20 µM (MedChem Express; Quimigen; Madrid, Spain) or vehicle (DMSO 0.5%) (Quimigen; Madrid, Spain) for 48 h. Viability was determined using a 3-(4,5-dimethylthiazol-2-yl)-2-5-diphenyltetrazolium bromide colorimetric assay (MTT #M2128; Sigma-Aldrich; Saint Louis, USA) which measures the conversion of the MTT to formazan, a compound that can be quantified by light

absorbance at a specific wavelength (490 nm) using a spectrophotometer (Varioskan flash reader; Thermo Fisher Scientific; Waltham, USA). Three experimental replicates for each treatment, or condition were made, in three or four biological replicates (each primary culture comes from a different gestation).

To detect and analyze GPX4 and xCT in WB total protein was isolated from the cortical cultures at different time points of maturation (3, 6, 10 and 13 DIV). Ten µg of protein were loaded to 4–15% precast polyacrylamide gel (Mini-PROTEAN TGX Precast Protein Gels; Bio-Rad; Hercules, USA) with a molecular weight marker (Precision Plus Protein™ Standards; Bio-Rad; Hercules, USA) and blotted onto PVDF-LF membranes (Millipore; Burlington, USA). Blocking, incubation and band measurements procedures were made as described in Material and methods section.

Table S1. Commercial proprietary predesigned qPCR forward and reverse primers obtained from IDT.

Gene	Encoded protein	Commercial primer name	Sequence
Trf	Endogenous transferrin	Mm.PT.58.23794874	forward: 5′-AGATAGAGTGTGAGTCAGCAGA-3′<br>reverse: 5′-TCTCGTAGTACTCTGCCATGA-3′
Aifm2	Ferroptosis Suppressor Protein 1	Mm.PT.58.30983091	forward: 5′-GACCTTCTCATCTCCACAAGC-3′<br>reverse: 5′- GCCTCTCTTCCACAGTTAACC-3′
Gpx4	Glutathione Peroxidase 4	Mm.PT.58.5454337	forward: 5′-CACTGTGGAAATGGATGAAAGTC-3′<br>reverse: 5′-CGCAGCCGTTCTTATCAATG-3′
Ftl1	Ferritin light chain	Mm.PT.58.32413506.g	forward: 5′-GACTTAGAGCAGCGCCTTG-3′<br>reverse: 5′-GAAGCGAGTACAGTGGGAATC-3′
Slc3a2	Solute Carrier Family 3 Member 2	Mm.PT.58.41156435	forward: 5′-ACCTCACTCCCAACTACCA-3′<br>reverse: 5′-CATTCATCAGCTTTCCCACATC-3′
Slc11a2	Divalent Metal Transporter 1	Mm.PT.58.16122997	forward: 5′-GCTTGCATCTTGCTGAAGTATG-3′<br>reverse: 5′-CATGTCAGAACCAATGATTGCC-3′
Tfrc	Transferrin receptor	Mm.PT.39a.22214833.g	forward: 5′-TCAAGCCAGATCAGCATTCTC-3′<br>reverse: 5′-AGCCAGTTTCATCTCCACATG-3′
Gapdh	Glyceraldehyde 3-phosphate dehydrogenase	Mm.PT.39a.1	forward: 5′-AATGGTGAAGGTCGGTGTG-3′<br>reverse: 5′-GTGGAGTCATACTGGAACATGTAG-3′
Pcbp2	Poly(RC) Binding Protein 2	Mm.PT.58.7663496	forward: 5′-CATTCCACAGCCAGATTTGAC-3′<br>reverse: 5′-CATGAGAAGTAGTTTGAGCAGATG-3′

Table S2. Antibodies used for WB and immunohistochemistry (IHC).

Primary antibody	Dilution	Method	RRID/Ref.	Company
Mouse anti-Transferrin receptor (H68.4) (TfR)	1:1000	WB	AB_2533029	Thermo Fisher Scientific
Mouse anti-glyceraldehyde-3-phosphate dehydrogenase (GAPDH)	1:2000	WB	AB_2536381	
Rabbit anti-ferritin light chain	1:1000	WB	AB_1523609	Abcam
Rabbit anti-Glutathione Peroxidase 4 (GPX4)	1:2000	WB	AB_10973901	
Rabbit anti-4 hydroxynonenal (4-HNE)	1:100	IHC	AB_722490	
Goat anti-mouse Transferrin (mTf)	1:1000	WB	AB_1147328	Novus Biologicals
Rabbit anti-xCT complex (SLC7A11 subunit – Solute Carrier Family 7 Member 11)	1:1000	WB	AB_2239445	
Mouse anti-Neuronal Nuclei (NeuN)	1:100	IHC	AB_2298772	Sigma-Aldrich
Mouse anti-NRAMP 2 (G-5), so called divalent metal transporter 1 (DMT1)	1:200	WB	AB_10610255	Santa Cruz Biotechnology
Mouse anti-human Tf (GMA-099)	1:250	WB	Lot#109052B	Green Mountain Antibodies
Rabbit anti-rat Tf	1:150	WB	Cat#55729	Cappel, ICN Pharmaceuticals
**Secondary antibody**	**Dilution**	**Method**	**RRID**	**Company**
IRDye-800CW donkey anti-mouse	1:25,000	WB	AB_2716622	Li-COR Bioscience
IRDye-680RD donkey anti-rabbit	1:15,000	WB	AB_2716687	
IRDye-680RD donkey anti-goat	1:10,000	WB	AB_2650427	
Donkey anti-rabbit IgG (H+L) Alexa Fluor 555	1:500	IHC	AB_162543	Thermo Fisher Scientific
Donkey anti-mouse IgG (H+L) Alexa Fluor 555	1:500	IHC	AB_141607	

Figure S1

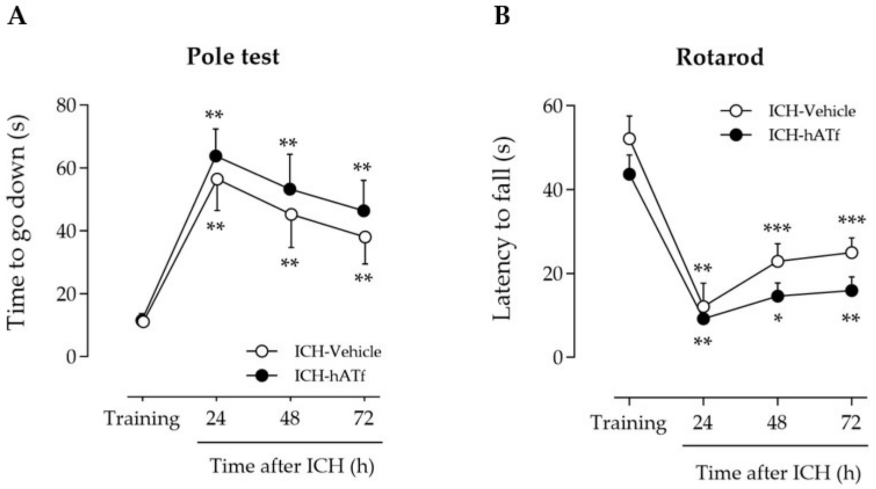

Figure S1. Effect of ICH and treatment with vehicle or hATf on the neurobehavioral performance of mice at 24, 48 and 72 h post-ICH induction in **(A)** the pole test and **(B)** the rotarod. *$p<0.05$, **$p<0.01$ and ***$p<0.005$ vs respective training (repeated measures one-way ANOVA and Tukey's test). Mean and SEM are shown.

Figure S2

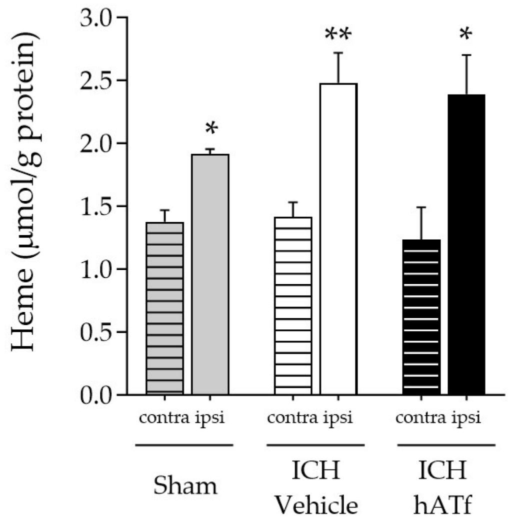

Figure S2. Heme levels in homogenates of the contralateral (contra) and the ipsilateral (ipsi) hemispheres of ICH mice treated with vehicle or hATf; a group of sham mice was included to determine the effect of the surgery associated to the administration of collagenase i.c.v.; *$p<0.05$ and **$p<0.01$ vs respective contra (paired *t* test).

Figure S3

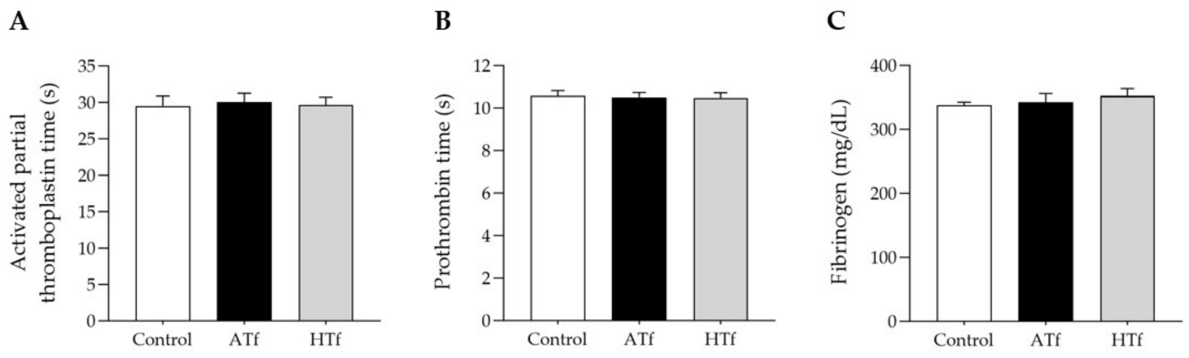

Figure S3. Effect of the addition of human ATf or human HTf on coagulation parameters of freshly obtained human blood: **(A)** activated partial thromboplastin time, **(B)** prothrombin time, and **(C)** fibrinogen. No significant differences were found (one-way ANOVA and Tukey's test).

Figure S4

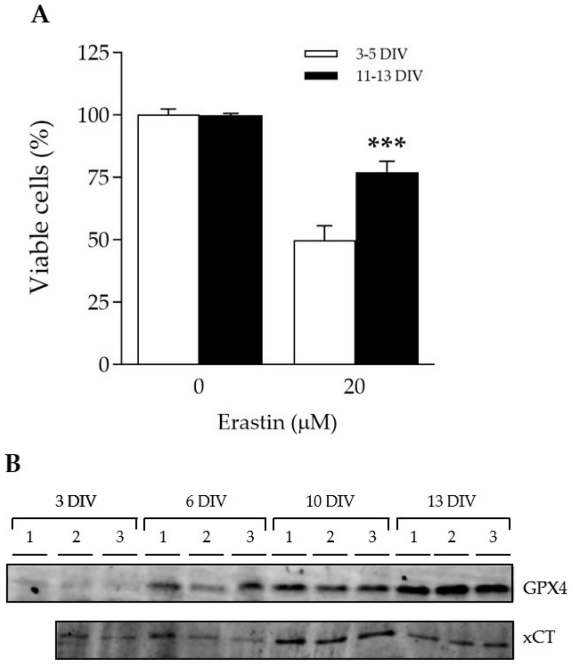

Figure S4. (A) Effect of the ferrostatin inducer erastin on neuronal viability *in vitro*. Young/undifferentiated (3-5 days-in-vitro/DIV) and differentiated (11-13 DIV) cell cultures were treated with 0 or 20 μM erastin for 48 h, and MTT levels were measured to determine % of viable cells. **(B)** Representative Western Blot images of triplicate wells showing the levels of glutathione peroxidase 4 (GPX4) and cysteine/glutamate antiporter xCT in undisturbed neuronal primary cell cultures at different days in vitro (DIV). ***p<0.005 vs 3-5 DIV (*t* test).

Figure S5

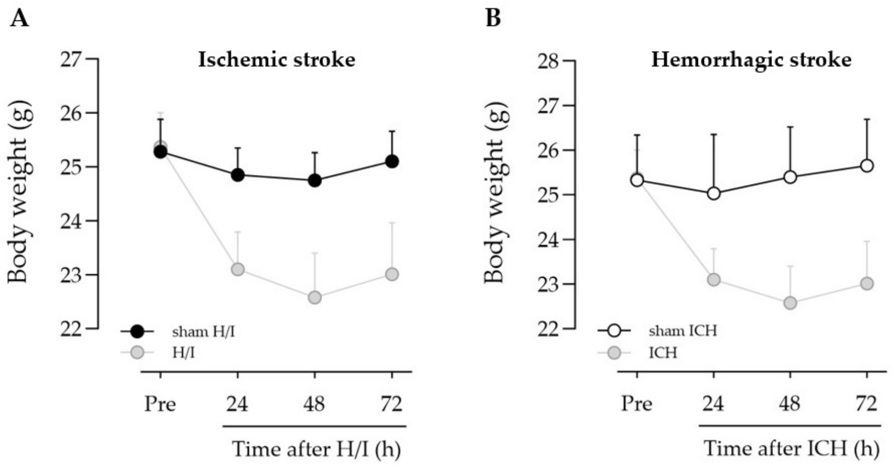

Figure S5. Body weight of **(A)** sham H/I (n=4) and **(B)** sham ICH (n=4) mice over the course of the experimental period; no statistically significant changes were observed. For the purpose of comparison, body weight loss of **(A)** H/I and **(B)** ICH mice are also depicted in pale gray.

Gel S1 (Figure 2C)

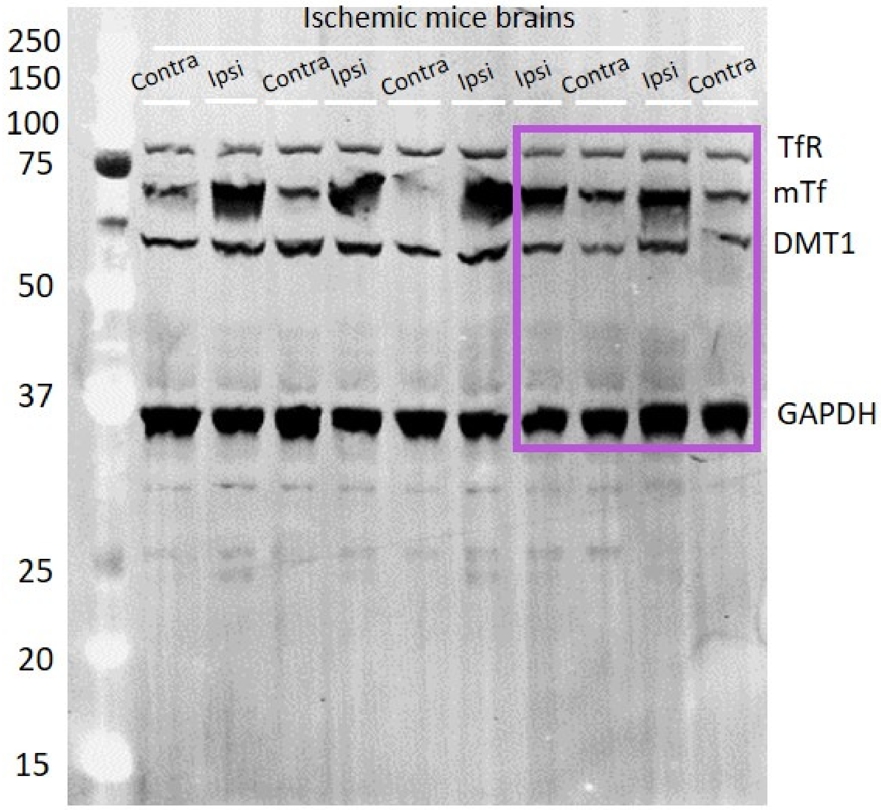

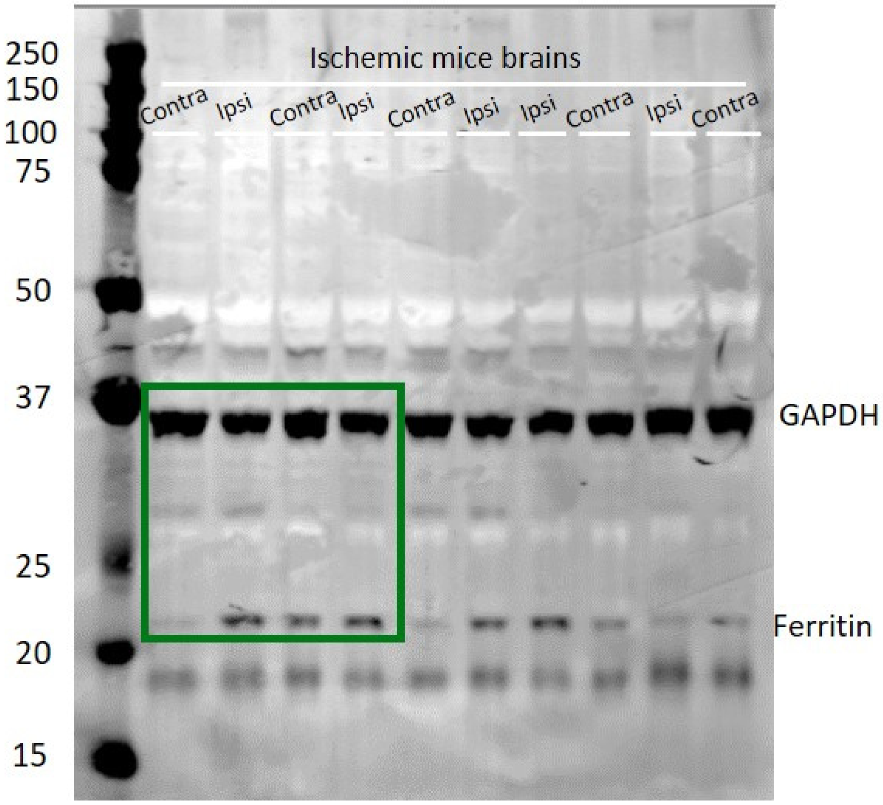

Gel S2 (Figure 2D)

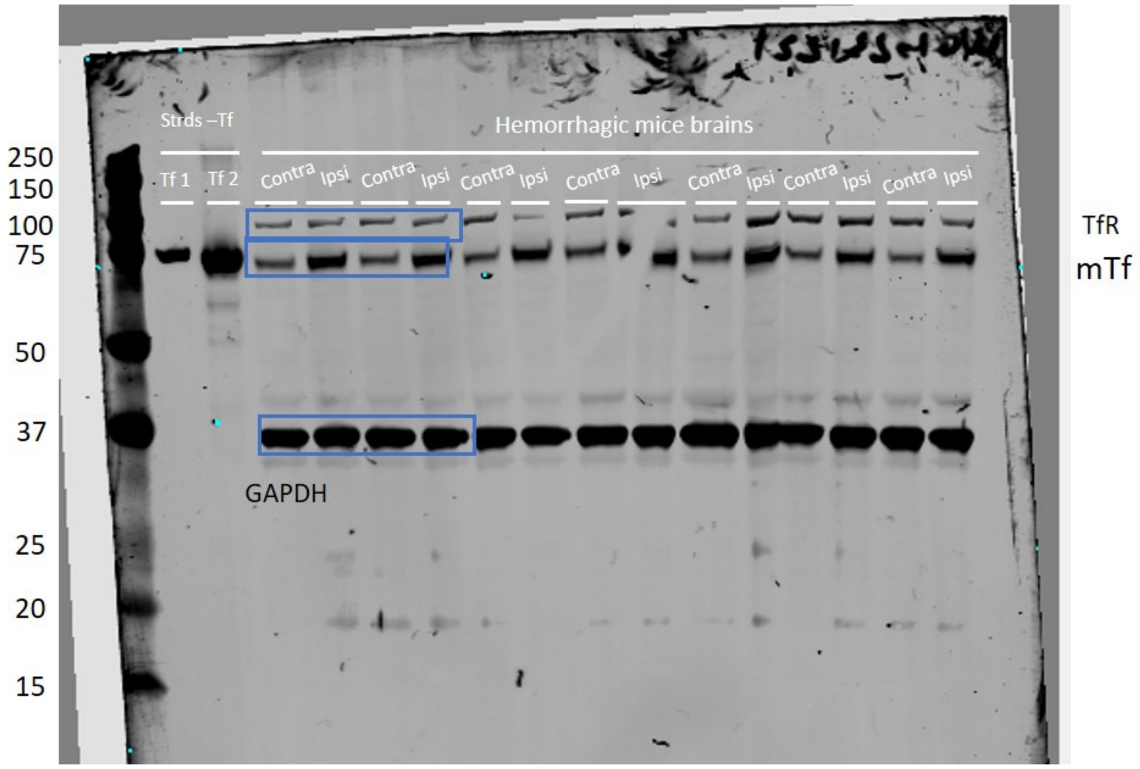

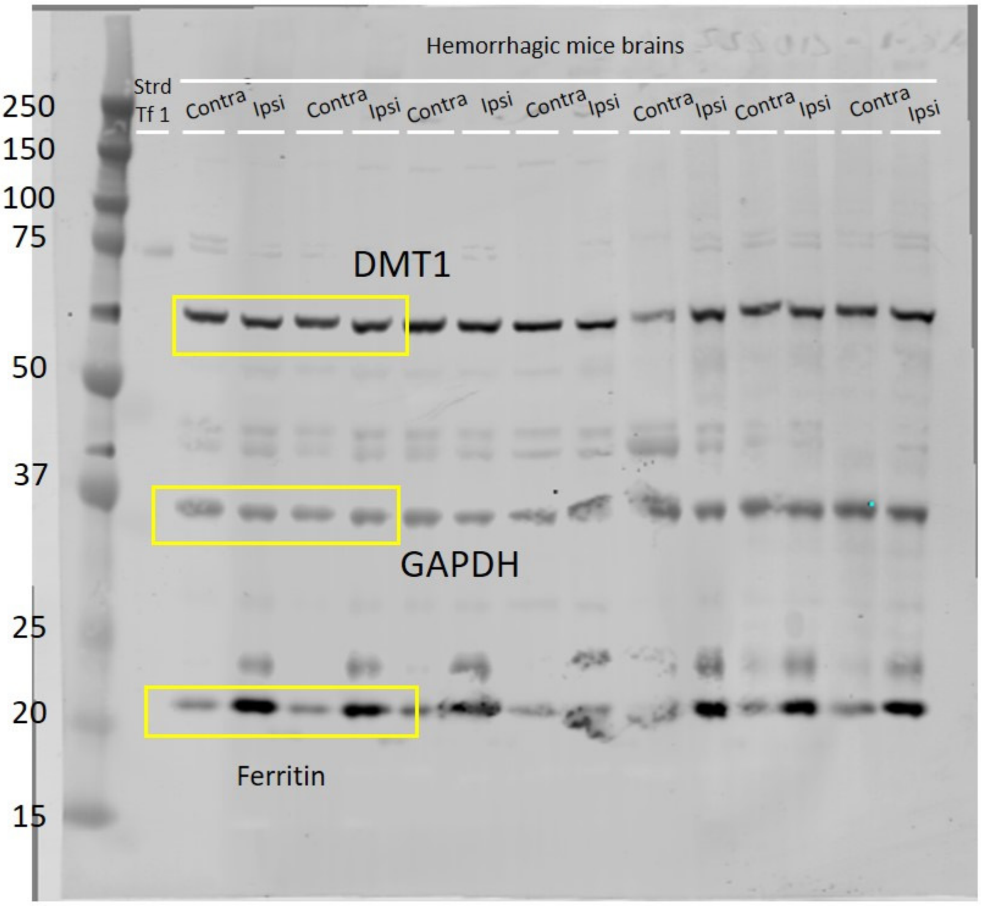

Gel S3 (Figure 3G)

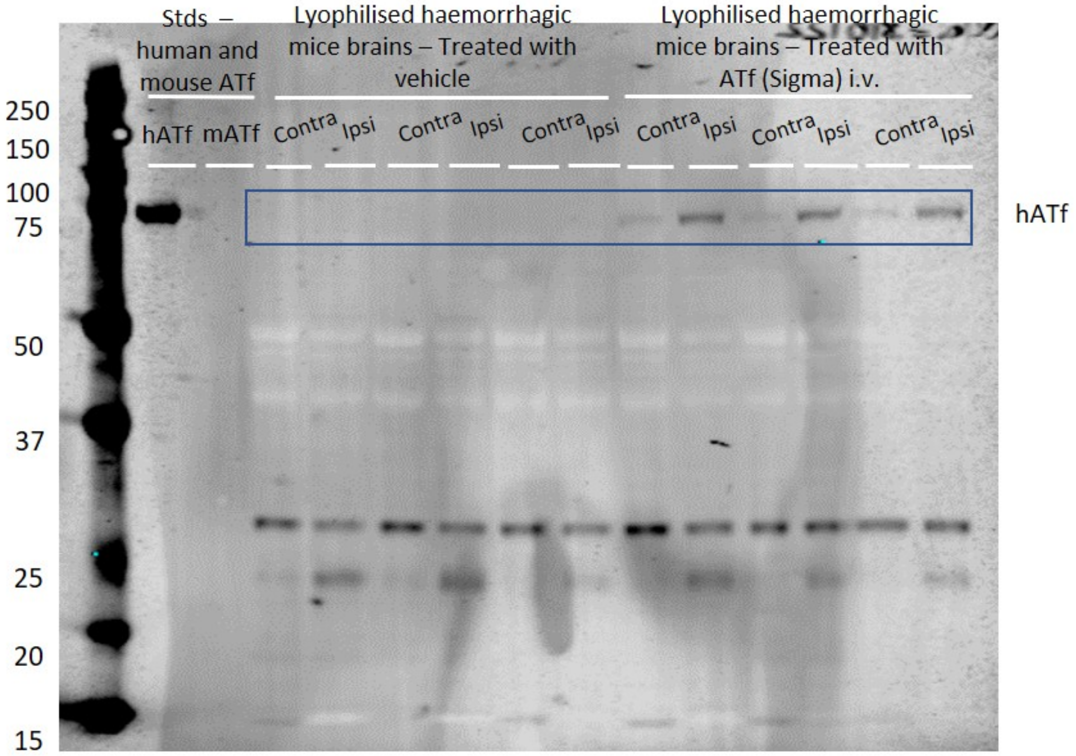

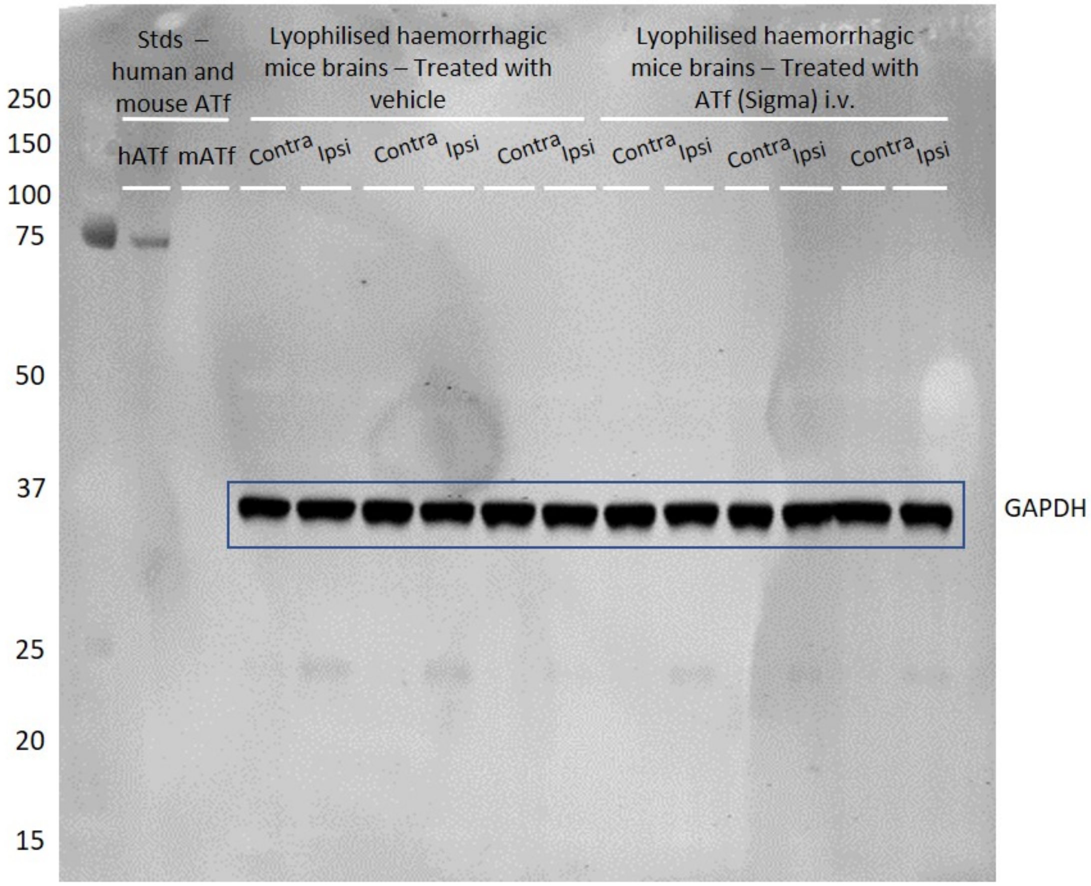

Gel S4 (Figure 5)

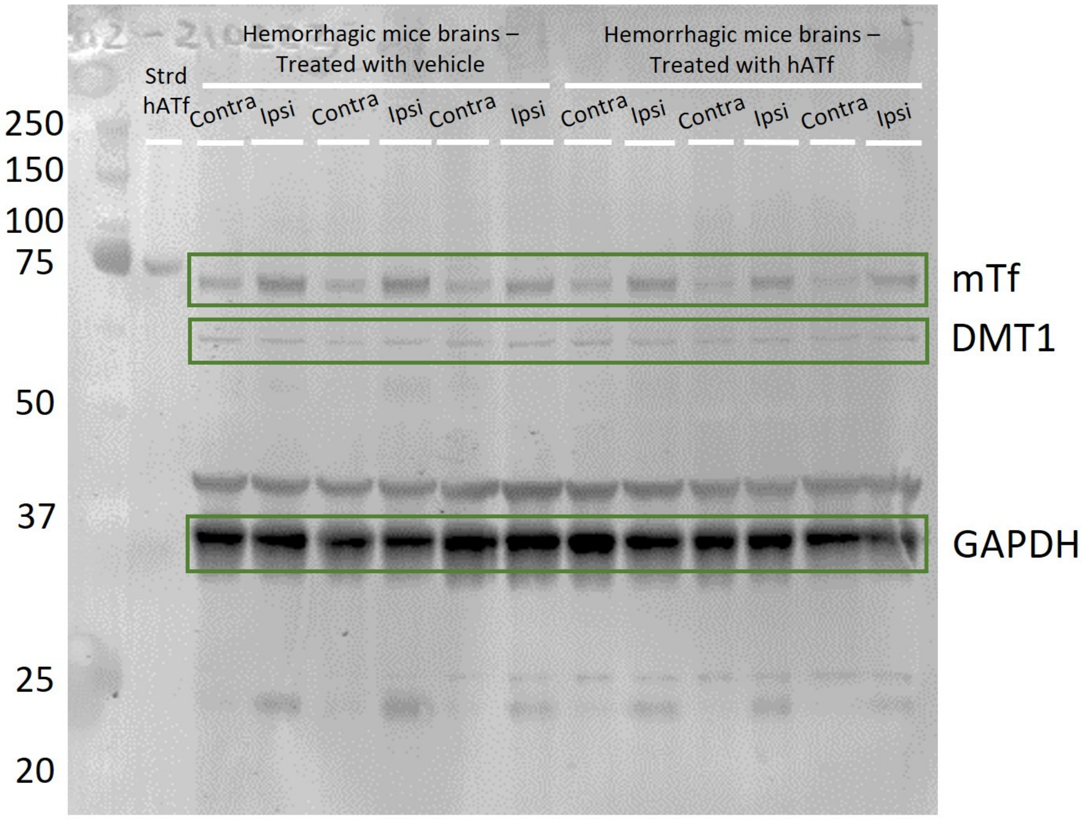

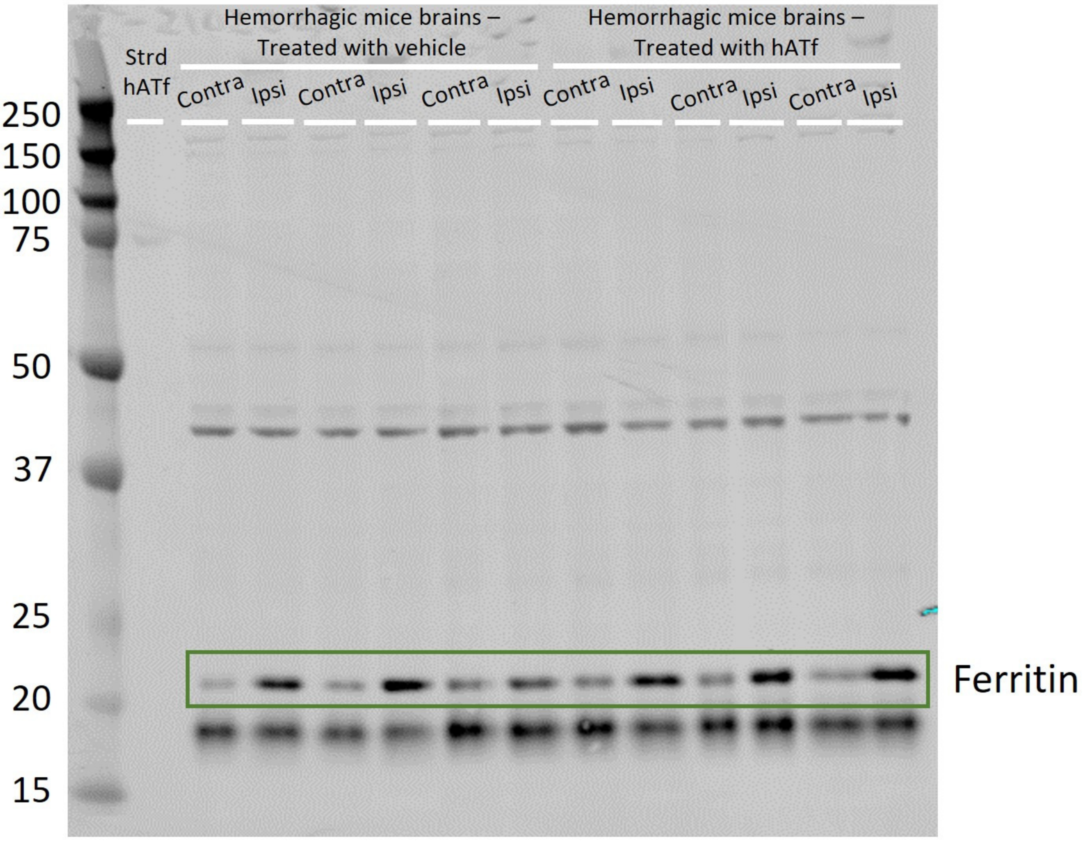

Gel S5 (Figure 6)

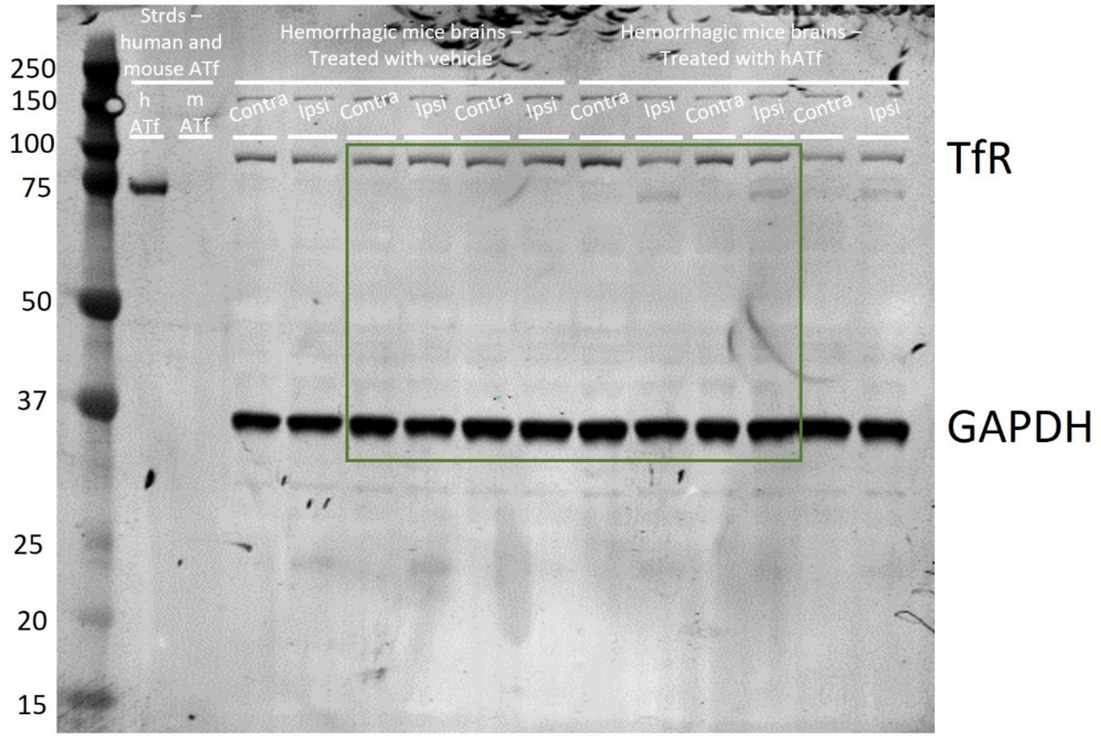

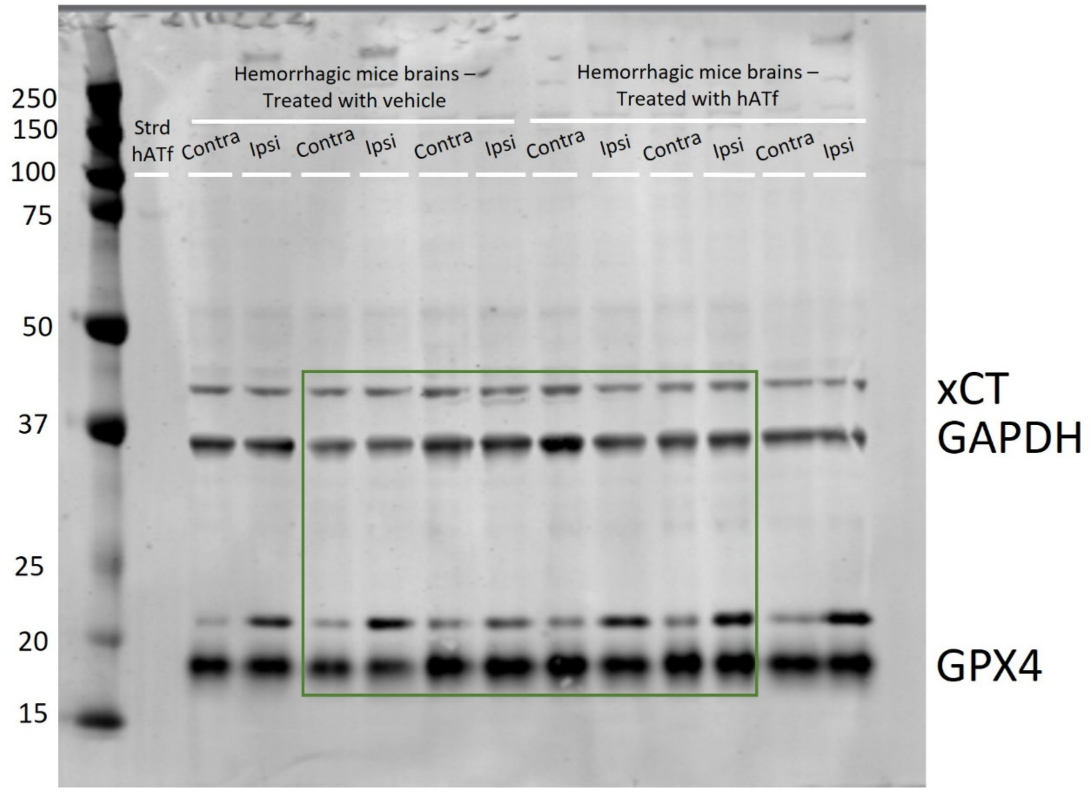

Gel S6 (Figure 8B)

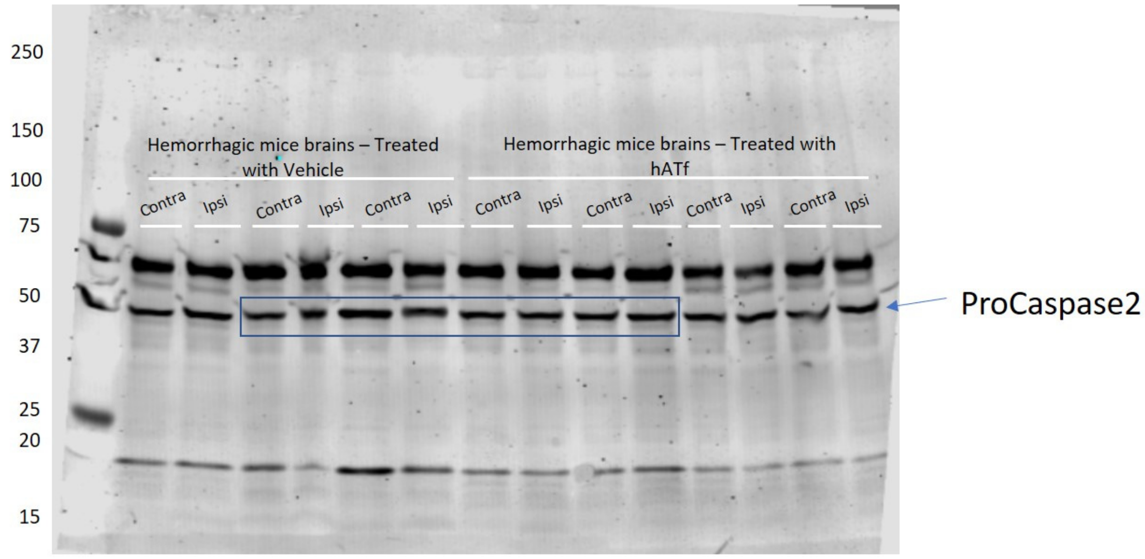

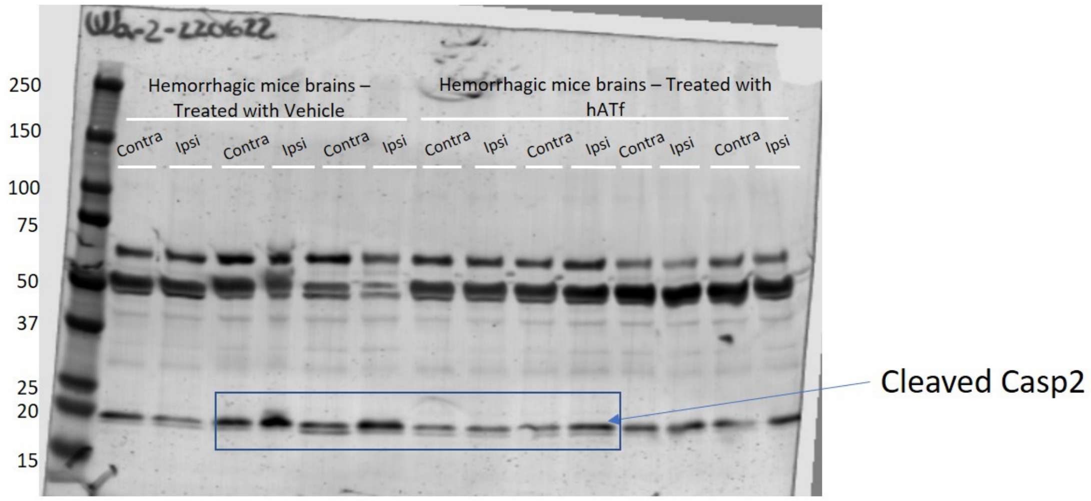

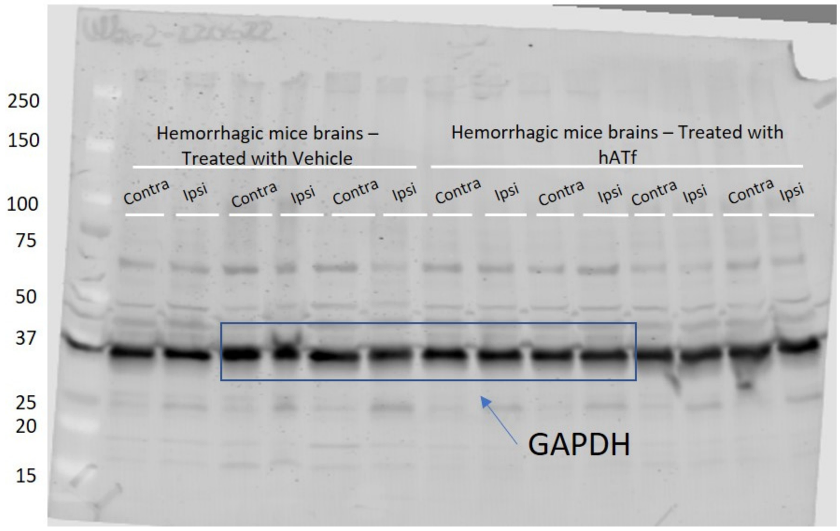

Supplement: Supplementary file 1 [file antioxidants-12-01945-s001.zip › antioxidants-2583369-supplementary.pdf]
